# Observations of the efficacy of the artificial lens cushion plate technique in hard-core cataract surgery

**DOI:** 10.3389/fmed.2024.1406578

**Published:** 2024-09-05

**Authors:** ZhiQing Huang, MiYun Zheng, MaoDong Xu, Lei Cai, XiaoQing Song

**Affiliations:** The First Hospital of Putian City, Putian, China

**Keywords:** IOL cushion plate technique, hard-core cataract, complications, corneal endothelial cells, central corneal thickness (CCT)

## Abstract

**Objective:**

To evaluate the efficacy of intraocular lens (IOL) cushion plate technology in reducing corneal endothelial cell loss during hard-core cataract surgery compared with conventional ultrasonic emulsification.

**Methods:**

Seventy-six patients with hard-core cataracts who underwent surgery at our institution from April 2019 to June 2022 were included. The patients were divided into an observation group (IOL cushion plate technology, 38 patients) and a control group (conventional ultrasonic emulsification, 38 patients). Surgical outcomes, including the corneal endothelial cell loss rate, best corrected visual acuity (BCVA), and central corneal thickness (CCTc), were compared between the two groups.

**Results:**

Preoperative patient characteristics were similar between the groups. Postoperatively, both groups demonstrated similar BCVA and CCTc values on days 7 and 30. However, compared with the observation group, the control group presented a significantly greater rate of corneal endothelial cell loss on postoperative days 7 and 30 (*p* < 0.05). Intraoperative complications and postoperative complications were notably greater in the control group (*p* < 0.05). The observation group had reduced ultramilk time and total energy consumption (*p* < 0.05).

**Conclusion:**

IOL cushion plate technology offers advantages in preserving corneal endothelial cells during hard-core cataract surgery, potentially improving surgical safety and efficacy.

## 1 Introduction

Cataracts are a common eye disease that occurs in the elderly population and requires surgical treatment. The surgical treatment of cataracts mainly involves suction removal of cloudy lenses and replacement of transparent an IOL to improve the clinical symptoms of patients and promote the recovery of their visual function. In the process of formulating the surgical plan, the actual situation of the patient needs to be fully considered, and one of the key concerns is the hardness of the cataract crystalline nucleus (1). Cataracts, specifically those classified as hard-core (grades IV ~ V), are associated with increased surgical complexity and risk. These risks include posterior capsule rupture, vitreous loss, and zonular dehiscence, among others. In the mature or overmature stage of senile cataracts, most hard-core cataract lens nuclei are dark yellow, dark tan or black, and visual dysfunction is severe. In the surgical treatment of hard-core cataract patients, ultrasonic emulsification is generally chosen as the treatment method, which often requires an increase in ultrasound energy during the operation, but there is a risk of intraocular tissue damage (2). To reduce the risk of surgical treatment, strengthening the protection of the cornea and posterior capsule is necessary, and the use of IOL cushion plate technology can reduce the ultrasound energy to a certain extent, prevent damage to the posterior capsule, and reduce corneal irritation, which in turn is conducive to rapid and good postoperative recovery (3). In this study, 76 hard-core cataract patients who underwent surgical treatment at our hospital from April 2019 ~ June 2022 were selected as research subjects to explore the impact of the application of IOL pad technology on surgical efficacy and patients’ postoperative recovery.

## 2 Information and methods

### 2.1 General information

The study included 76 patients with hard-core cataracts who underwent surgical treatment at our institution between April 2019 and June 2022. Using the random number table method, the participants were divided into an observation group and a control group, with 38 individuals in each group. Table 1 presents the general patient information. Patients with hard-core cataracts, categorized as grade IV ~ V density according to the Emery-Little classification, met the criteria for inclusion in this research.

**Table 1:** General information of the two groups of patients.

Group	*n*	Age	Sex (m/f)	Nuclear classification (grade 4/5)	Preoperative visual acuity	
					<0.1	≥0.1
Observation group	38	68.34	13/25	30/8	8	30
Comparison group	38	71.84	15/23	29/9	5	33
χ^2^/t		0.385	0.226	0.076	0.835	
P		0.894	0.634	0.783	0.361	

### 2.2 Methods

Surgical equipment: An Infiniti ultrasonic emulsifier made by American Alcon Company, German Zeiss OPMI Lumera T microscope was used.

Surgical procedure: Proparinamine hydrochloride eye drops were applied before surgery, ocular surface anesthesia was applied (1 ~ 2 drops/min, 2 ~ 3 times), and main and side incisions were made at 11:00 and 2:00 at the angle of the scleral margin. A viscoelastic agent was injected into the anterior chamber. If necessary, the anterior capsule membrane was stained with Taipan orchid, and continuous circular tearing of the capsule was performed. The capsule was separated from the cortical water, and an ultrasonic emulsification head was placed in the anterior chamber, penetrating the suprapapillary head deep enough toward the center of the nucleus to fix the lens nucleus and perform intercepted cleavage of the nucleus.

#### 2.2.1 Observation group

**Figure 1:**
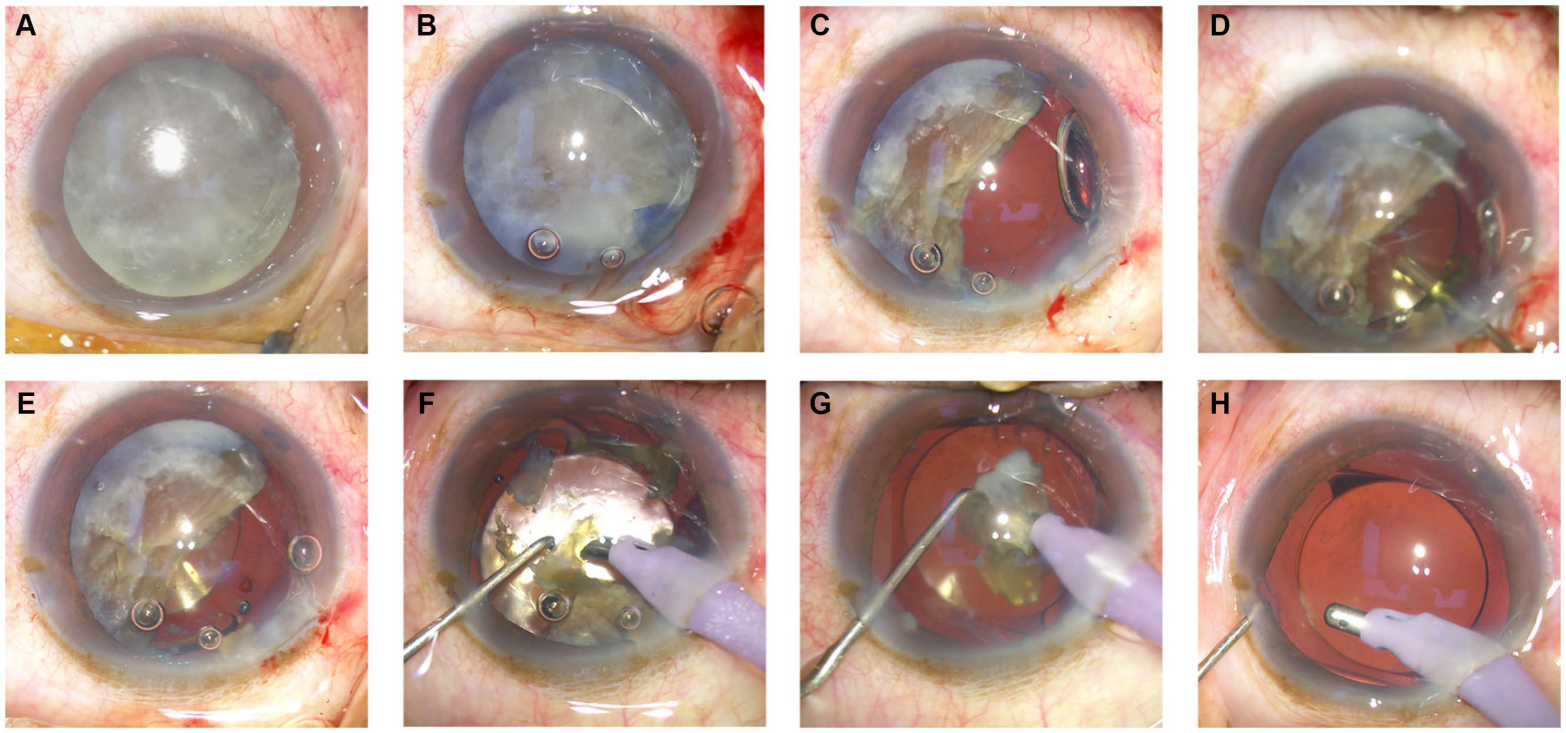
Overview of the artificial lens cushion plate technique.

Before emulsification of the remaining half of the nuclear fragment (Figure 1A), a cohesive viscoelastic material was carefully injected beneath the nuclear fragment (Figure 1B). This action gently nudged the fragment in the direction opposite the corneal incision (Figure 1C). A foldable intraocular lens (IOL) was subsequently inserted into the well-inflated capsular bag, which was positioned posteriorly to the nuclear fragment (Figures 1D,E). Finally, the remaining piece of the nuclear fragment was emulsified and extracted from within the capsular bag (Figures 1F–H).

#### 2.2.2 Control group

The nucleus was initially segmented into four parts via the phaco-chop technique. These nuclear segments were subsequently emulsified one after the other through ultrasonication. In the absence of a cortical shell within the capsular bag, meticulous care was exercised during emulsification of the final nuclear segment, ensuring that it was positioned more anteriorly, between the iris plane and the anterior chamber, to facilitate the procedure. After ultrasonic emulsification of all the nuclei, a viscoelastic agent was injected into the capsular bag, and the IOL was implanted.

Finally, the crystal cortex was aspirated, the viscoelastic agent was removed, the mouth was closed with water, and the eye was wrapped with Dengbisu ophthalmic ointment.

### 2.3 Observation indices

① Treatment effect: The best corrected visual acuity (BCVA) was detected while a corneal endothelial cytometer (Solvay SW-7000 type) was used, and the corneal endothelial count (ECCr) value was determined. Anterior segment optical correlation tomography (Zeiss Cirrus HD-OCT 4000 type) was applied to measure the central corneal thickness (CCTc), the changes at 7 d and 30 d after surgery were compared, and the surgical efficacy was evaluated. ② Complications: The occurrence of iris injury, corneal edema, uveitis, posterior capsular rupture and other complications was observed during and after surgery.

### 2.4 Statistical processing

SPSS 21.0 statistical software was used to process and analyze the data, and the measurement data are expressed as the mean ± sd, which conformed to a normal distribution and was assessed by the t value. The count data are expressed as (%), which was tested by the χ^2^ test, and *p* < 0.05 indicated that the difference was statistically significant.

## 3 Results

### 3.1 Postoperative recovery of hard-core cataract patients in the two groups

**Figure 2:**
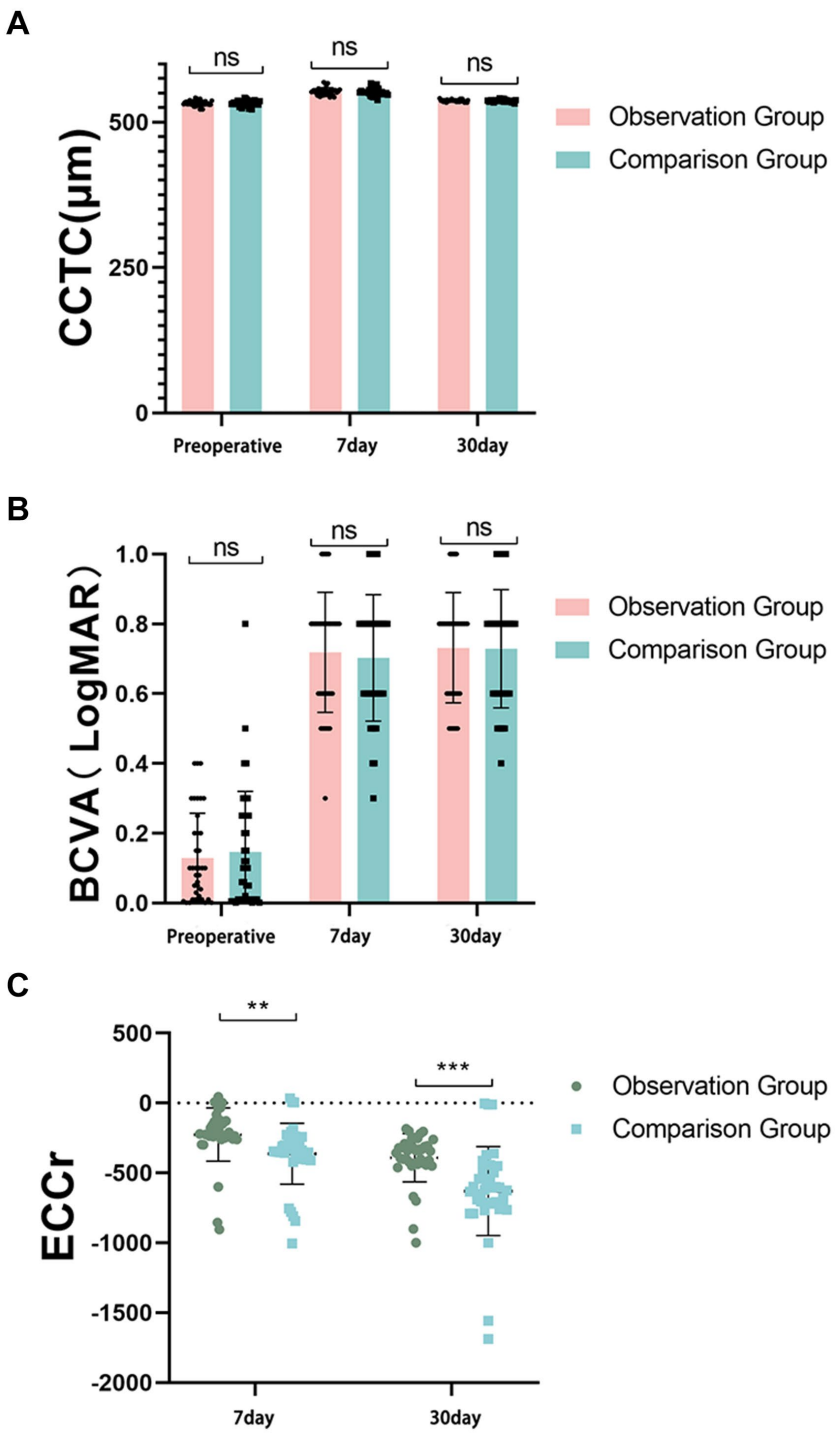
Observations of postoperative recovery in two groups of patients with sclerocorneal cataracts. **(A)** The central corneal thickness (CCTc) was not significantly different between the two groups. **(B)** The best corrected visual acuity (BCVA) was not significantly different between the two groups. **(C)** The ECCr in the control group was greater than that in the observation group on postoperative day 7 post-operation, **p = 0.0045 < 0.005. The ECCr in the control group was greater than that in the observation group on day 30 post-operation, ***p = 0.0001 < 0.001.

The central corneal thickness (CCTc) and best corrected visual acuity (BCVA) were examined on days 7 and 30 after surgery, and no statistically significant differences in the CCTc and BCVA indices were detected between the two groups (Figures 2A,B). In addition, detection of the corneal endothelial cell loss rate (ECCr) revealed that the ECCr of the control group was significantly greater than that of the observation group on the 7th postoperative day (*p* =0.0045 < 0.005), and the ECCr of the control group was significantly greater than that of the observation group on the 30th postoperative day (*p* =0.0001 < 0.001) (Figure 2C).

### 3.2 Intraoperative and postoperative complications in patients with hard-core cataracts in both groups

During the intraoperative and postoperative periods, we observed complications such as iris injury, corneal edema, uveitis, and posterior capsule rupture. We found that the complication rate in the control group was greater than that in the observation group, and the difference in the complication rate between the two groups during the intraoperative and postoperative periods was statistically significant (*p* < 0.05). For detailed information on both groups, please refer to Table 2.

**Table 2:** Intraoperative and postoperative complications in two groups of patients with sclerotic cataracts [*n* (%)].

Group	Iris damage	Corneal edema	Rupture of the posterior capsule membrane	Complications
Observation group (*n* = 38)	0 (0)	1 (2.63)	0 (0)	1 (2.63)
Comparison group (*n* = 38)	1 (2.63)	4 (10.52)	1 (2.63)	6 (15.79)
χ^2^				3.934
*p* value				<0.05

As shown in Table 2, the complication rates for various issues (iris damage, corneal edema, and rupture of the posterior capsule membrane) in the observation group were lower than those in the control group. The observation group had only 1 case of corneal edema, with an overall complication rate of 2.63%, whereas the control group had 1 case of iris damage, 4 cases of corneal edema, and 1 case of rupture of the posterior capsule membrane, resulting in an overall complication rate of 15.79%. Statistical analysis indicated that the difference in complication rates between the two groups was significant (χ^2^ = 3.934, *p* < 0.05).

### 3.3 Comparison of energy application

**Figure 3:**
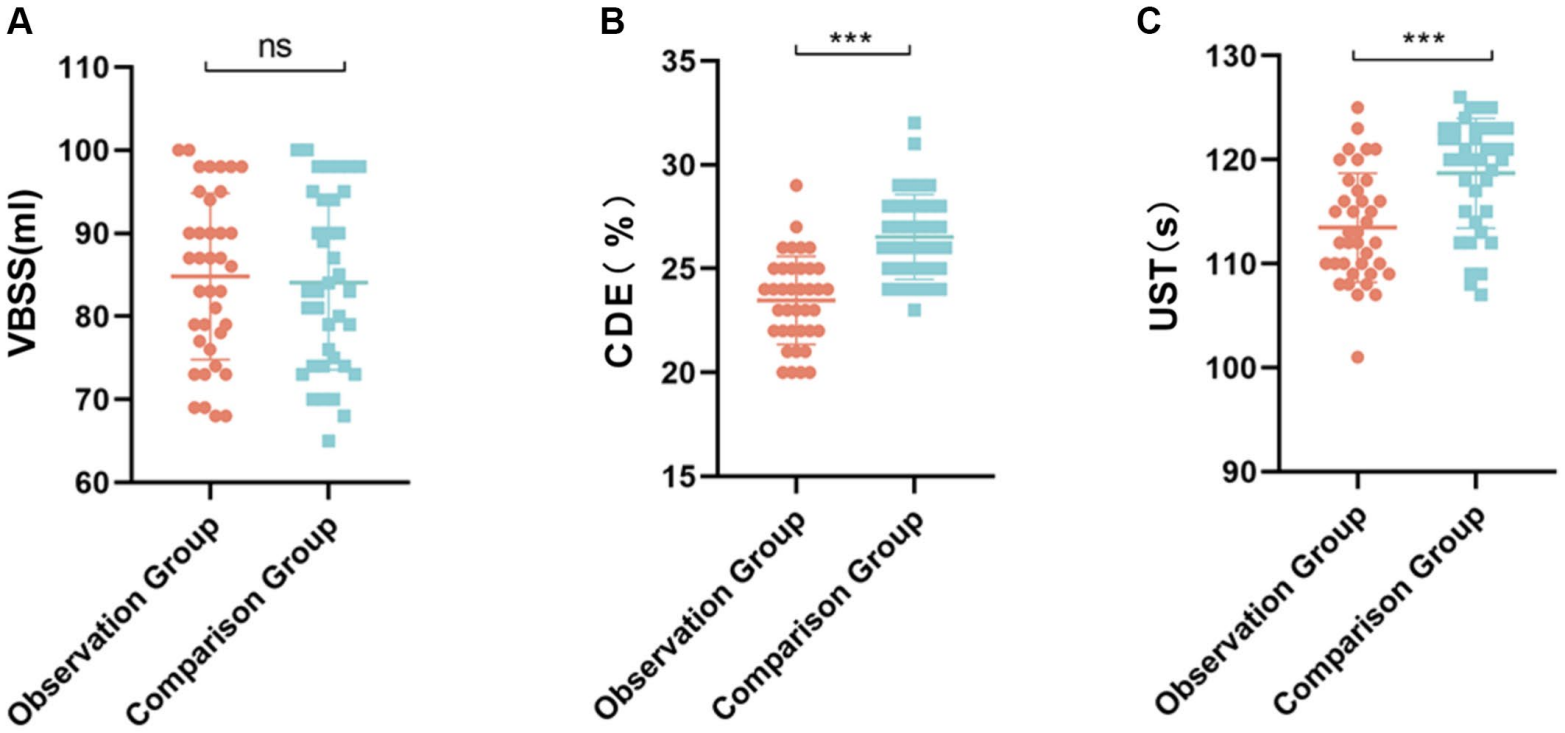
Intraoperative energy application in the two groups of patients. **(A)** The total volume of balanced salt solution used was not significantly different between the two groups, ns *p* > 0.05. **(B)** The phacoemulsification energy consumption of the observation group was significantly lower than that of the control group, ***p < 0.001. **(C)** The observation group had a significantly shorter phaco time than did the control group, ***p < 0.001.

The difference in the total amount of balancing fluid between the two groups was not statistically significant (*p* > 0.05) (Figures 3A,B), the intraoperative total amount of ultra milk time in the observation group was less than that in the control group, the total energy consumed by the ultra milk in the observation group was less than that in the control group, and the difference between the two groups was statistically significant (*p* < 0.05), as shown in Figure 3C.

## 4 Discussion

The success rate of ultrasonic emulsification for hard-core cataracts is limited because of patient characteristics, including large hard lens cores, poor capsule elasticity, fragile suspensory ligaments, and low corneal endothelial cell counts (4). The posterior capsule is thinner than the anterior capsule, making capsular bag collapse likely (5). Intraoperative mechanical trauma and turbulent flow can damage the corneal endothelium and lens posterior capsule, resulting in serious complications, such as posterior capsule rupture, vitreous detachment, and nucleus drop into the vitreous cavity (6). Postoperative complications include corneal edema and compensatory failure, as well as drug-induced side effects such as dry eye syndrome (7). To overcome these problems in hard-core cataract surgery, we modified traditional ultrasonoemulsification by implanting an IOL before complete removal of the lens nucleus instead of stepwise removal of the lens material followed by ultrasonic emulsification until all lens material was removed.

In this study, in the comparison of the BCVA, ECCr and CCTc, the rate of corneal endothelial cell loss in the observation group was lower than that in the control group at 30 d after surgery. Compared with the control group, the observation group had fewer complications, such as iris damage, corneal edema, and uveitis during and after surgery; the total ultrasonic emulsification time of the observation group was less than that of the control group during surgery; and the total energy consumed by ultrasonic emulsification in the observation group was less than that in the control group. It is suggested that the surgical operation be carried out in accordance with the conventional ultrasonic emulsification surgical treatment process, during which the IOL cushion plate technique is applied to lower the position of ultrasonic emulsification and provide protection for the posterior capsule membrane (8). In the process of surgical treatment, ultrasound energy can be reduced to decrease the stimulation of the corneal endothelium and avoid a reduction in corneal endothelial cells. The application of IOL cushion plate technology has a good effect on maintaining corneal atrial water barrier function and preventing corneal edema (9).

IOL cushion plate technology was first used in wrong or unsatisfactory IOL replacement surgery when the IOL is used as a pad but not in the process of nuclear emulsification (10). Luo et al. (1), Parkash et al. (8), and Hua et al. (11) applied this technique to nuclear phacoemulsification in hard nucleus cataracts and confirmed that this technique can effectively protect the posterior capsule of the lens, which is consistent with our conclusion. In a randomized study by Luo et al. (1), 80 dense cataract cases were split into two interventions. In Group I, the IOL was placed after complete nucleus removal, whereas in Group II, it was inserted prior to removing the last nuclear quarter. No significant disparity in corneal thickness between groups was observed at any measurement. Notably, Group II demonstrated less corneal endothelial cell depletion on days 7 and 30 post-surgery. Specifically, on day 7, cell loss averaged 10.29% in Group II versus 14.37% in Group I (*p* < 0.05). By day 30, these values were 16.88 and 23.32%, respectively (*p* < 0.05). Furthermore, the initial day’s mean CCT was markedly lower in Group II than in Group I (13.50% vs. 19.42%, *p* < 0.05) (1). Parkash et al. (8) discussed how the intraocular lens (IOL) acts as a scaffold, offering the benefit of a stable barrier or support over the undamaged posterior capsule. When there is stable support over the relaxed posterior capsule, the use of an IOL scaffold in a Morgagnian cataracts can prevent posterior capsule rupture and prevent the complications associated with it. This method also stabilizes the capsular bag by allowing it to expand during surgery (8). In the study by Hua et al. (11), 12 patients with Morgagnian cataracts underwent modified IOL implantation in the capsular bag post-capsulorhexis. In three hypermature cases with small, rigid nuclei, the IOL was inserted directly after complete capsulorhexis, shielding the posterior capsule during phacoemulsification. In the remaining nine cases, which had larger, softer nuclei, the IOL was placed after partial nuclear emulsification. All procedures were successful, with no complications to the posterior capsule or vitreous loss, indicating effectiveness in protecting the posterior capsule during phacoemulsification (11).

Phacoemulsification surgery in cases of hard cataracts can lead to endothelial damage through several mechanisms: extended phacoemulsification time and increased ultrasound energy use, air bubbles and localized temperature elevations, mechanical trauma from instrument insertion, and collisions with lenticular debris, particularly swirling debris (12–16). Additionally, biochemical stressors such as oxidative damage from free radicals produced during ultrasonic energy application have been identified (17). Consequently, we speculate that the IOL cushion plate technology may protect the corneal endothelium by gently pressing the nucleus down with the posterior capsule shielded by the artificial lens cushion plate during surgery, increasing the depth of the anterior chamber to facilitate remaining phacoemulsification within the capsular bag. The specific mechanism might involve the deepened anterior chamber, which not only distances the phaco tip from the corneal endothelium, thereby reducing direct or indirect damage from energy and temperature, but also decreases the likelihood of swirling lenticular debris coming into contact with the corneal endothelium. In our study, the application of IOL cushion plate technology resulted in a significant reduction in corneal endothelial cell loss rate on the 7th and 30th postoperative days within the observation group compared to the control group.

Regarding the shorter phacoemulsification time and lower energy consumption observed in the observation group, these differences could be attributed to the modified surgical technique that, which allows for a more efficient emulsification process. The cushioning effect of the IOL may facilitate nucleus fragmentation, thereby reducing the need for prolonged ultrasonic energy application. This improved efficiency may not only increase the safety of the procedure by reducing thermal and mechanical stress on intraocular tissues but also may minimize the risk of potential complications such as corneal burn or capsular rupture.

The complication rate in our study did not significantly differ between the observation and control groups. However, the types of complications, their clinical management, and the implications for patient recovery are critical aspects to consider. For example, while the rate of posterior capsular rupture is low, it remains a severe complication that can lead to further surgical challenges and affect visual outcomes. Future studies should aim to provide a more comprehensive analysis of complication profiles and their management to better understand the clinical implications of IOL cushion plate technology.

The surgical effects were evaluated via BCVA and CCTc measurements. The BCVA serves as a direct indicator of a patient’s visual function post-surgery, whereas the CCTc provides information on corneal integrity and potential edema, which can indirectly impact visual acuity. The findings of our study suggest that both the BCVA and CCTc are valuable metrics for assessing surgical success and patient recovery, reinforcing the importance of multifaceted outcome evaluations in cataract surgery research.

In our study, the use of IOL cushion plate technology resulted in a lower corneal endothelial cell loss rate on the 7th and 30th postoperative days in the observation group than in the control group. This reduction in endothelial cell loss is likely attributable to the protective barrier provided by the IOL cushion, which minimizes direct contact and mechanical stress on the corneal endothelium during phacoemulsification. The reduced ultrasonic energy and time required for nucleus emulsification with the IOL cushion plate technology also contributed to this protective effect. By preserving the integrity of the corneal endothelium, this technique may enhance long-term corneal health and visual acuity, underscoring the importance of endothelial cell preservation in cataract surgery outcomes. In our clinical work, we found that IOL cushion plate technology is not limited to the treatment of hard-core cataracts but can be used to create padding before removing the lumpy and flocculent cortex that is tightly adherent to the posterior capsule during routine cataract echo capillarization, which reduces the probability of aspiration of the anterior posterior capsule during the process of removal and prevents rupture of the posterior capsule membrane. In addition, in some special cases, such as high intraocular pressure leading to significant corneal edema, insufficient corneal endothelium, and poor corneal endothelial cell function, to avoid further damage to the corneal endothelium during surgery, IOL cushion plate technology can be applied, which would require a large amount of data to be confirmed in the clinic.

We acknowledge the limitations of our study, including the small sample size, its retrospective design, and the lack of long-term follow-up data. These factors may limit the generalizability and completeness of our findings. In this study, the maximum observation time was only 1 month, and we were unable to compare the occurrence of postoperative cataracts between the two groups. The implantation of the IOL first hinders the complete removal of the cortex to a certain extent, and the step of “posterior capsular polishing” cannot be implemented, so whether there is any effect needs to be further explored. To address these limitations, future prospective studies with larger cohorts and extended follow-up periods are necessary to validate the benefits of IOL cushion plate technology and assess its long-term impact on patient outcomes, including the development of PCO and other late-onset complications. Additionally, further research should explore the nuances of individual patient differences and device efficiency to refine surgical techniques and optimize patient care.

In summary, the application of IOL pad technology during ultrasonic emulsification has obvious advantages for the treatment of patients with hard-core cataracts. The intraoperative surgical operation is simple and can reduce the amount of ultrasound energy and time used during ultrasonic emulsification. In addition, the application of IOL pad technology can increase the depth of the anterior chamber for intracapsular ultrasonic emulsification, which can be far from the cornea and greatly reduces the chances of damaging the corneal endothelium. Moreover, the implantation of the IOL first protects the posterior capsule more reliably. The posterior capsule is more reliably protected after IOL implantation, which creates favorable conditions for the successful completion of cataract surgery.

## Data Availability

The original contributions presented in the study are included in the article/Supplementary material, further inquiries can be directed to the corresponding author.
